# Effect of repaglinide versus glimepiride on daily blood glucose variability and changes in blood inflammatory and oxidative stress markers

**DOI:** 10.1186/1758-5996-6-54

**Published:** 2014-05-05

**Authors:** Masahiro Yamazaki, Goji Hasegawa, Saori Majima, Kazuteru Mitsuhashi, Takuya Fukuda, Hiroya Iwase, Mayuko Kadono, Mai Asano, Takafumi Senmaru, Muhei Tanaka, Michiaki Fukui, Naoto Nakamura

**Affiliations:** 1 Department of Endocrinology and Metabolism, Kyoto Prefectural University of Medicine Graduate School of Medical Science, 465 Kajii-cho, Kamigyo-ku, Kyoto 602-8566, Japan

**Keywords:** Cardiovascular disease, Continuous glucose monitoring, MAGE, Glimepiride

## Abstract

**Background:**

Hemoglobin A1c is the main treatment target for patients with type 2 diabetes. It has also been shown recently that postprandial glucose and daily glucose fluctuations affect the progression of diabetic complications and atherosclerotic damages.

**Methods:**

Continuous glucose monitoring was performed in patients with type 2 diabetes to evaluate the efficacy of repaglinide vs. glimepiride on postprandial glucose spikes and fluctuations. A total of 10 Japanese patients with type 2 diabetes treated with glimepiride monotherapy were enrolled. After observation period for 8 weeks, glimepiride was changed to repaglinide. Continuous glucose monitoring was performed whilst consuming calorie-restricted diets for two days at baseline and at the end of the 12-week trial. Blood and urine samples were collected for measurement of glucose control parameters and inflammatory and oxidative stress markers on the last day of taking either glimepiride or repaglinide.

**Results:**

Nine patients completed the trial. Although the glucose control parameters were not significantly different between glimepiride and repaglinide, the mean amplitude of glycemic excursions measured by continuous glucose monitoring was significantly reduced by changing treatment from glimepiride to repaglinide. The levels of plasminogen activator inhibitor-1, high sensitivity C-reactive protein, and urinary 8-hydoroxydeoxyguanosine were reduced significantly by repaglinide treatment.

**Conclusion:**

These results suggest that repaglinide may decrease the risk of cardiovascular disease in type 2 diabetes by minimizing glucose fluctuations thereby reducing inflammation and oxidative stress.

## Background

Inflammation and oxidative stress have emerged as important factors in atherosclerosis, and have therefore attracted clinical attention as novel risk factors for cardiovascular diseases (CVD). Some studies in patients with diabetes have shown that serum inflammation markers, such as high-sensitivity C reactive protein (hs-CRP), interleukin-6 (IL-6), interleukin-10 (IL-10) and tumor necrosis factor-α (TNF-α), and oxidative stress markers, such as urinary 8-hydoroxydeoxyguanosine (u-8-OHdG) and 8-iso-prostaglandin F (2α) (u-8-isoPGF_2α_), were associated clearly with future coronary events [1-5].

Hemoglobin A1c (HbA1c) is the main target for patients with type 2 diabetes. However, postprandial glucose levels have been shown to be closely associated with increased CVD [6]. It has been reported that activation of either inflammation or oxidative stress by postprandial hyperglycemia is an important mechanism in the pathogenesis of atherosclerosis [7]. Glucose fluctuations during postprandial periods have a more specific triggering effect on oxidative stress than chronic sustained hyperglycemia [8]. Therefore, acute glucose swings should be targeted as an anti-atherosclerotic strategy in the treatment of type 2 diabetes.

Sulfonylurea agents (SUs) such as glimepiride are used widely to treat patients with type 2 diabetes, although they do not reduce glucose swings. On the other hand, repaglinide, a meal-time insulin secretagogue approved for treatment of type 2 diabetes, reduces postprandial blood glucose (PBG) peaks, lowers 24-h blood glucose (BG) profiles, and reduces HbA1c levels [9-11]. Animal experiments have demonstrated that repaglinide also has an anti-oxidative effect [12]. There is also evidence that progression of carotid intima-media thickness in patients treated with repaglinide is significantly attenuated compared with treatment with SUs [13,14]. These findings suggest that repaglinide has anti-inflammatory and anti-oxidative effects by reducing glucose fluctuations. The mean amplitude of glycemic excursions (MAGE) calculated from continuous glucose monitoring (CGM) is the gold standard for evaluating glucose variability. However, no studies have demonstrated differences between the effects of repaglinide and SUs on glucose fluctuations based on data obtained from CGM devices.

In this study, we assessed the effects of repaglinide treatment on PBG peaks and glucose fluctuations using CGM, and compared changes in oxidative stress and inflammation markers with those measured during glimepiride treatment.

## Subjects and methods

### Study subjects and protocol

The subjects were recruited from the outpatient clinic at Hospital of Kyoto Prefectural University of Medicine between July 2012 and October 2013. The subjects were patients with type 2 diabetes older than 20 years. All the patients were treated with a stable dosage of glimepride only and had stable glycemic control (stable HbA1c levels between 7.0 ~ 8.0% for at least 8 weeks before inclusion). Patients with severe chronic diabetic complications, heart failure, liver dysfunction, renal dysfunction, inflammatory diseases, or malignancies were excluded. All recruited patients with major cardiovascular event history were excluded from this study. The purpose and risks of the study were explained to all the subjects, and informed consent was obtained prior to enrollment. The study was approved by the Ethical Committee of the Kyoto Prefectural University of Medicine.

**Figure 1:**
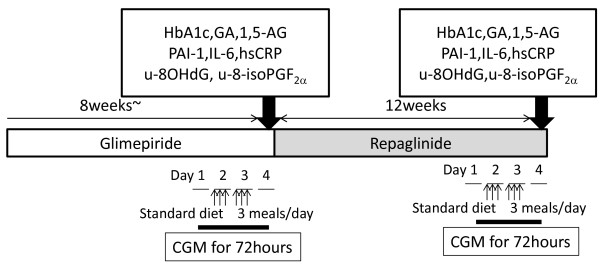
**Study protocol.** The patients were treated with glimepiride monotherapy for 8 weeks to achieve glycemic control. After changing treatment to repaglinide, the patients were monitored for 12 weeks to ensure they had stable glucose control. Blood and urine samples were collected, and CGM was performed at the end of both treatment periods.

The study protocol is summarized in Figure 1. After recruitment, all the patients were treated with glimepiride monotherapy for 8 weeks under normal daily life conditions, followed by the first CGM. The dosage of glimepiride was not changed until the first CGM was completed. The first CGM was performed for 3 days (72 hours). On the second and third days of monitoring, the patients had calorie-adjusted meals delivered to their homes (mealtime; FUNDERY Co., Ltd., Tokyo, Japan) that consisted of a breakfast, lunch and dinner (440 ~ 510 kcal, protein 18 g, fat 9 g, carbohydrate 52 ~ 67 g in each meal). On the last day of CGM, fasting blood samples were collected for measurement of fasting blood glucose (FBG), HbA1c, glycoalbumin (GA), 1,5-anhydroglucitol (1,5-AG), and insulin. The same samples were used to measure inflammatory markers including plasminogen activator inhibitor-1 (PAI-1), IL-6, and hs-CRP. U-8-OHdG and u-8-isoPGF_2α_ were measured in spot urine samples collected at the same time. The day after the first CGM, glimepiride was changed to repaglinide as follows: 0.5 mg, 1.0 mg and 2.0 mg of glimepiride once a day in the morning were changed to 0.125 mg, 0.25 mg, and 0.5 mg of repaglinide 3 times a day just before meals, respectively. After 4 weeks of repaglinide treatment the patients had blood tests to confirm they had no side effects and stable glycemic control. After 12 weeks of repaglinide treatment, a second CGM was performed using the same protocol as the first CGM. Blood and urine tests were also performed as described above.

### Analytical methods

Plasma glucose was determined by a standard laboratory assay. Plasma insulin, C-peptide, and IL-6 were analyzed by CLEIA, and HbA1c was assayed using high-performance liquid chromatography and expressed as National Glycohemoglobin Standardization Program Units. GA, 1,5-AG, u-8-OHdG, and u-8-iso PGF_2α_ were analyzed by EIA, and PAI-1 by the LPIA-tPAI test (SRL, Tokyo, Japan).

CGM was performed using a i-Pro2 digital recorder (MiniMed, Medtronic, Northridge, CA, USA). MAGE, the standard deviation (SD) of blood glucose level, mean blood glucose level (mean BG), percent of hours under 3.9 mmol/L of serum glucose in 24 hours and percent of hours over 10.0 mmol/L of serum glucose in 24 hours were calculated from the glucose curve on day 3 in order to exclude the influence of the meals on the first day of CGM.

### Statistical methods

Data in the tables and figures were expressed as mean ± SD, with the exception of CGM (mean ± SEM). The effects of changing treatment were analyzed using paired *t* test. All the statistical analyses were performed using PRISM5 software (GraphPad Software, Inc., CA, USA).

## Results

### Clinical characteristics at baseline

Ten patients were enrolled in the study, with a total of 9 patients completing the trial. One patient was withdrawn because of gastrointestinal symptoms caused by repaglinide. All the patients were Japanese with a mean age of 68.0 ± 6.5 years and HbA1c of 7.0 ± 0.2% (range 6.7 ~ 7.2%). The estimated duration of diabetes was 18.3 ± 12.8 years (range 5 ~ 44 years). The mean dose of glimepiride was 1.0 ± 0.4 mg/day. Six of the participants were treated with anti-hypertensive agents; two with angiotensin II receptor blocker (ARB) only;two with Ca blocker only, and; two with ARB and Ca blocker. One participant was treated with acetyl salicylic acid. Any changes of anti-hypertensive, anti-platelet, and anti-lipid agents during the current study were not done. The other baseline data are shown in Table 1 (glimepiride treatment group).

**Table 1:** Comparison the clinical and laboratory markers at glimepiride treatment and repaglinide treatment

	**Glimepiride**	**Repaglinide**	***p***
Number (male)	9 (7)	9 (7)	-
Age (years)	68.0 ± 6.5	-	-
Duration (years)	18.3 ± 12.8	-	-
Body weight (kg)	64.3 ± 12.7	64.2 ± 12.3	0.86
Body mass index (kg/m^2^)	24.7 ± 3.7	24.7 ± 3.7	0.91
Dose (mg)	1.0 ± 0.4	0.75 ± 0.32	-
sBP (mmHg)	132.6 ± 4.2	126.2 ± 3.8	0.42
dBP (mmHg)	74.6 ± 4.0	70.6 ± 3.8	0.47
HbA1c (%)	7.0 ± 0.2	7.0 ± 0.2	0.62
FBS (mmol/L)	7.8 ± 1.9	7.7 ± 1.5	0.78
GA (%)	17.2 ± 1.7	17.3 ± 1.3	0.84
1,5-AG(mg/ml)	7.1 ± 3.3	7.1 ± 3.6	0.96
IRI (mU/ml)	6.0 ± 2.6	5.2 ± 3.6	0.41
s-CPR(mg/dl)	2.3 ± 1.9	1.6 ± 0.7	0.17
HOMA-IR	2.4 ± 1.7	2.1 ± 1.1	0.17
HOMA-β	33.7 ± 16.3	30.9 ± 15.8	0.15

Data are expressed as means ± SD. sBP, systolic blood pressure; dBP, diastolic blood pressure; HbA1c, hemoglobin A1c; FBS, fasting blood glucose; GA, glycoalbumin; 1,5-AG, anhydroglucitol; IRI, immunoreactive insulin; s-CPR, serum C-peptide immunoreactivity.

### The effects on body weight, BMI, and glycemic control

After 12 weeks of treatment with repaglinide, there were no significant changes in body weight or BMI (Table 1). None of the glucose control parameters (HbA1c, GA, and 1,5-AG) were changed by repaglinide treatment. Homeostasis model assessment indices of insulin resistance (HOMA-IR) and β-cell function (HOMA-β) were comparable with glimepiride or repaglinide treatment (Table 1).

### The effects on parameters of daily glucose change

**Figure 2:**
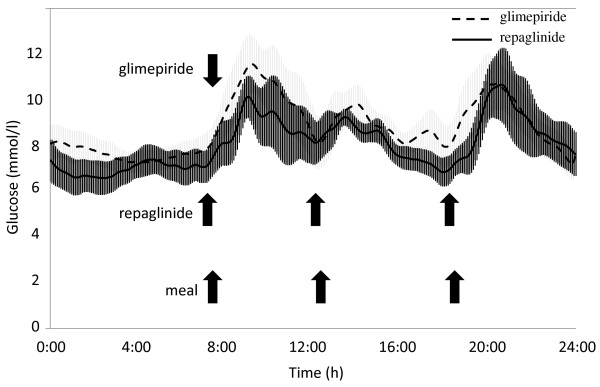
**Blood glucose profiles measured by CGM during glimepiride or repaglinide treatment (n = 8).** Glimepiride was taken once a day in the morning, while repaglinide was taken three times a day before every meal. The blood glucose profiles on day 3 are shown for all patients. The effects of the meal with unrestricted calories consumed on day 1 and 2 were excluded from the analyses. Blight shade area shows mean ± SEM of serum glucose levels during glimepiride treatment. Dark shade area shows mean ± SEM of serum glucose levels during repaglinide treatment.

The 24-h blood glucose profiles in day 3 of CGM are shown in Figure 2. One of the nine patients declined to undergo CGM. In the remaining eight patients, the SD for blood glucose levels and mean BG were similar between the two treatments (Table 2). In contrast, MAGE, a marker of daily glucose fluctuations, decreased significantly with repaglinide treatment (Table 2. glimepiride, 108.0 ± 55.5 vs. repaglinide, 65.1 ± 30.4; *P* < 0.05). The mean percent of hours over 10.0 mmol/L of serum glucose in 24 hours at repaglinide treatment was significantly lower than that at glimepiride treatment (Table 2. glimepiride, 31.3 ± 27.1% vs. repaglinide, 10.1 ± 10.5%; *p* < 0.05).

**Table 2:** Parameters calculated by CGM during glimepiride or repaglinide treatment

	**glimepiride**	**repaglinide**	***p***
Max-BG(mmol/L)	13.5 ± 2.9	12.7 ± 3.6	0.51
Min-BG(mmol/L)	5.6 ± 0.9	5.3 ± 1.3	0.58
Max-Min (mmol/L)	7.9 ± 2.8	7.4 ± 3.4	0.63
SD (mmol/L)	1.9 ± 0.8	1.8 ± 1.0	0.78
Mean BG(mmol/L)	8.7 ± 1.6	8.0 ± 1.5	0.33
MAGE(mmol/L)	6.0 ± 3.1	3.6 ± 1.7	<0.05
Hours of under 3.9 mmol/L (% 24 hours)	0	0	-
Hours of over 10.0 mmol/L (% 24 hours)	31.3 ± 27.1	10.1 ± 10.5	<0.05

Data are expressed as means ± SD. BG, blood glucose; Max-Min, difference between Max-BG and Min-BG; SD, standard deviation; MAGE, mean amplitude of glycemic excursions.

### The effects on markers of blood inflammation and urine oxidative stress

**Figure 3:**
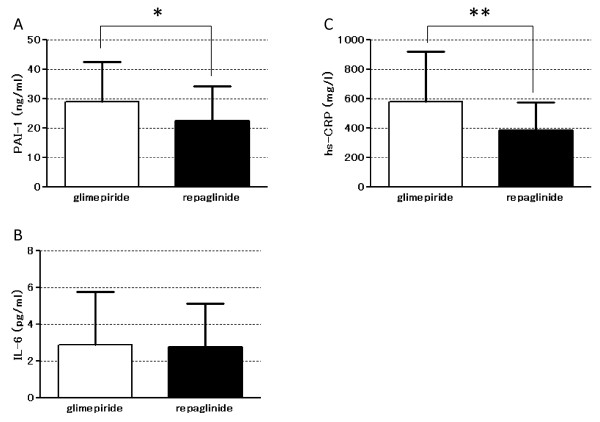
**Inflammation markers measured by in blood samples collected during treatment with either glimepiride or repaglinide. (A)** PAI-1, **(B)** IL-6, and **(C)** hs-CRP levels are shown (n = 9). *p < 0.001; **p < 0.05.

The levels of plasma PAI-1 (Figure 3. glimepiride, 28.9 ± 13.5 vs. repaglinide, 22.5 ± 11.6; *P* < 0.05) and hs-CRP (Figure 3. glimepiride, 580.3 ± 339.7 vs. repaglinide, 382.3 ± 191.1; *P* < 0.05) were decreased significantly following repaglinide treatment compared with glimepiride treatment. On the other hand, there was no significant change in plasma IL-6 levels.

**Figure 4:**
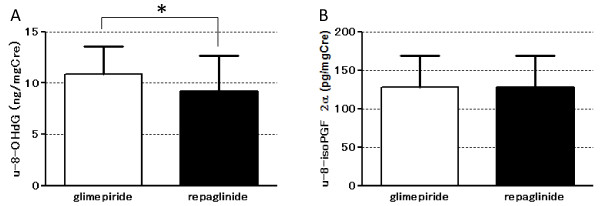
**Oxidative stress markers measured in urine samples collected during treatment with either glimepiride or repaglinide. (A)** u-8-OHdG and **(B)** u-8-isoPGF_2α_ levels are shown (n = 9). *p < 0.05.

U-8-OHdG and u-8-isoPGF_2α_ were measured to assess the effect on oxidative stress. The level of u-8OHdG was decreased significantly with repaglinide treatment (Figure 4. glimepiride, 10.9 ± 2.7 vs. repaglinide, 9.2 ± 3.5; *P* < 0.05), whereas u-8-isoPGF_2α,_ was not.

## Discussion

This study on patients with type 2 diabetes on glimepiride showed that a change in treatment to repaglinide resulted in a significant decrease in both inflammatory (PAI-1, hs-CRP) and oxidative stress markers (u-8-OHdG), despite glucose control parameters (HbA1c, GA, and mean BG) remaining unchanged. We also demonstrated that repaglinide improved postprandial glucose excursions and MAGE assessed by CGM following calorie-adjusted meals.

Repaglinide is an insulin secretagogue agent with a more rapid anti-hyperglycemic action and a shorter duration than SUs, and therefore provides better control of postprandial hyperglycemia [15,16]. In this study, the smaller fluctuations in blood glucose levels with repaglinide treatment were consistent with the pharmacodynamic properties of this agent.

The mean level of glycemia assessed by HbA1c is a well known risk factor for the development of diabetic vascular complications. However, recent studies have suggested that blood glucose variability is more deleterious than a constant high glucose as it accelerates diabetic macrovascular complications [17]. Xiao-min et al. reported that parameters of glycemic variability calculated from CGM data, such as SD and MAGE, were increased as atherosclerosis progressed in patients with type 2 diabetes, and that increases in carotid intima-media thickness correlated significantly with MAGE [18]. Furthermore, accumulated data have indicated there is a significant relationship between glucose fluctuations and oxidative stress that may lead to the development of atherosclerosis. A recent study demonstrated that chronic blood glucose variability induced chronic inflammation as assessed by blood hs-CRP levels, a marker that is recognized as a significant predictor of cardiovascular events [19]. Based on these reports, we assume that the smaller fluctuations in glucose levels we observed following repaglinide treatment compared with glimepiride treatment may have decreased the level of inflammation and oxidative stress biomarkers. These data raise the possibility that long-term repaglinide treatment may be beneficial for preventing macrovascular complications in type 2 diabetes.

Rizzo et al. also demonstrated using data from a meal-test that repaglinide was more efficient than glimepiride for controlling postprandial glucose excursions and reducing oxidative stress and inflammation markers. The duration of chronic sustained hyperglycemia and the acute fluctuations in glucose levels are two factors that contribute to diabetic complications. The measurement of MAGE is strongly recommended for predicting incident cardiovascular events [20,21]. CGM is the only method that allows estimation of a variety of glucose fluctuation parameters including MAGE. It has also been reported that oxidative stress markers (u-8-isoPGF_2α,_ serum 8-OHdG) and chronic inflammation marker (hs-CRP) correlate positively with MAGE, but not with HbA1c or FBS [22]. The present study confirmed the benefit of repaglinide treatment by incorporating detailed analysis of acute glucose fluctuations using CGM and simultaneous measurements of inflammation and oxidative stress markers under normal daily living conditions. Furthermore, the percent of hours over 10.0 mmol/L of serum glucose calculated by CGM at repaglinide treatment was lower than that at glimepiride treatment. This shorter exposure of hyperglycemia might contribute to decrease of inflammatory and oxidative stress markers.

The NAVIGATOR study, a large international placebo-controlled trial, did not demonstrate the validity of nateglinide (a short-acting insulin secretagogue) for reduction of the incidence of diabetes and cardiovascular events in the patients of impaired glucose tolerance [23]. It may be argued that this finding is contradictable with our current results predicting the suppressive effect of repaglinide, another short-acting insulin secretagogues, on inflammation and oxidative stress in type 2 diabetes patients. However, it should be noted that, in the NAVIGATOR study, the nateglinide treatment group was associated with greater body weight gain and failure of suppression of postprandial hyperglycemia. Therefore, we cannot refer to the significance of suppression of post-prandial hyperglycemia from the results of NAVIGATOR study. In addition, it has been reported that repaglinide has the stronger glucose lowering effect than nateglinide [24].

Many of the limitations stem from our sample size, which limits our statistical power to observe associations, although we conducted this study with the well controlled protocol and the selected subjects with the well-designed inclusion/exclusion criteria. We assumed that the other factors than the anti-diabetics, such as habitual and environmental factors, didn’t change during three months of the intervention period. In general, three months of the intervention period is considered to be appropriate for estimation of the merely drug effect. Actually, we confirmed that the patients’ physical conditions and lifestyles didn’t change every 4 week. Furthermore, setting the control group would help elucidating the mechanism underlying the drug effect.

## Conclusion

Repaglinide treatment is more beneficial than glimepiride for decreasing glucose variability in patients with type 2 diabetes. These smaller glucose fluctuations are associated with decreased levels of inflammation and oxidative stress markers. This change is important as it has the potential benefit of reducing CVD risk in patients with type 2 diabetes.

## Competing interests

The authors declare that they have no competing interests.

## Authors’ contributions

SM, KM, TF, HI researched data, MY wrote the manuscript. MA, TS, MT, MF, NN contributed to discussion, GH and MK reviewed/edited manuscript. All authors have approved the final version of this manuscript.

## Authors’ information

Masahiro Yamazaki is the first author and Goji Hasegawa contributed as senior author.
